# Dissecting the logical types of network control in gene expression profiles

**DOI:** 10.1186/1752-0509-2-18

**Published:** 2008-02-19

**Authors:** Carsten Marr, Marcel Geertz, Marc-Thorsten Hütt, Georgi Muskhelishvili

**Affiliations:** 1 Computational Systems Biology Group, Jacobs University, Campus Ring 1, 28759 Bremen, Germany; 2 Molecular Genetics Group, Jacobs University, Campus Ring 1, 28759 Bremen, Germany; 3 Institute for Bioinformatics and Systems Biology, Helmholtz Zentrum München – German Research Center for Environmental Health, 85764 Neuherberg, Germany

## Abstract

**Background:**

In the bacterium *Escherichia coli *the transcriptional regulation of gene expression involves both dedicated regulators binding specific DNA sites with high affinity and also global regulators – abundant DNA architectural proteins of the bacterial nucleoid binding multiple sites with a wide range of affinities and thus modulating the superhelical density of DNA. The first form of transcriptional regulation is predominantly pairwise and specific, representing digitial control, while the second form is (in strength and distribution) continuous, representing analog control.

**Results:**

Here we look at the properties of effective networks derived from significant gene expression changes under variation of the two forms of control and find that upon limitations of one type of control (caused e.g. by mutation of a global DNA architectural factor) the other type can compensate for compromised regulation. Mutations of global regulators significantly enhance the digital control, whereas in the presence of global DNA architectural proteins regulation is mostly of the analog type, coupling spatially neighboring genomic loci. Taken together our data suggest that two logically distinct – digital and analog – types of control are balancing each other.

**Conclusion:**

By revealing two distinct logical types of control, our approach provides basic insights into both the organizational principles of transcriptional regulation and the mechanisms buffering genetic flexibility. We anticipate that the general concept of distinguishing logical types of control will apply to many complex biological networks.

## Background

One important objection to Lamarckian evolution by inheritance of acquired characteristics emphasized by Bateson over forty years ago is the reduction of adaptational flexibility upon progressive specialization, necessitating the occurrence of genotypic changes compensating for this limitation [1]. In unicellular organisms such as bacteria, in keeping with Batesons' prediction the same acquired mutations beneficial in one environment can be restrictive in another [2]. At the same time, evolving *Escherichia coli *populations can demonstrate remarkable flexibility in genetic adaptation [3]. The mechanisms sustaining this flexibility remain unclear. In order to understand the genetic flexibility it is essential to decipher the organizational logic of transcriptional control. For the classical model organism *E. coli *the largest electronically accessible network integrating the data on the transcriptional regulation of genes is available [4]. The interlinked elements form a complex structure, which is essentially of digital nature (digital refers here to the fact that the network provides static information on the connections between unique, discontinuous components [5], e.g. a particular pair of regulating and regulated gene). Notably, such pair-wise connections are not necessarily reflected in genomic expression profiles [6,7] indicating that not all the interactions given in the network occur at all times. Furthermore, this type of network does not account for the analog mode of gene regulation via alterations of DNA topology – a long known control mechanism revived by recent DNA microarray analyses [8-10] (analog refers here to the fact that the expression of specific genes is under the control of continuous information provided by spatial distributions of supercoiling energy in the genome [11]). Indeed, transcriptional responses to alterations of DNA superhelicity reveal non-trivial spatial patterns, raising new questions on the coordination of genomic transcription [9,11] and the interplay between chromosomal organization and patterns in gene expression is now becoming the focus of computational analyses [12,13]. From these considerations it is obvious that a holistic theory of transcriptional regulation has to include the relationships between these two logically distinct (digital-binary and analog-continuous) types of information and therefore has to distinguish them in the first place. Although other mechanisms of gene regulation between the binary and continuous extremes can be considered, for understanding the organizational principles of transcriptional regulation we assume a working model here in which the impacts of the two distinct logical types of control – one of digital and another of analog type – are to be clearly distinguished and related to each other.

In the following, we will translate the patterns in gene expression changes observed under systematic variation of the two types of control into effective networks and study their connectivity. The effective networks are derived as subnetworks of two larger (static) networks: (1) the transcriptional regulatory network based upon the action of dedicated transcription factors; (2) spatial proximity of two genes on the circular chromosome.

We will statistically compare the properties of these effective networks with those obtained by random sampling of the static networks with a certain number of expression changes. The core quantity derived from these comparisons is the ratio of connected to isolated nodes (control ratio) and, furthermore, its z-score with respect to the random networks. This z-score we denote the confidence level of the particular control type (control type confidence, CTC).

## Results

**Figure 1:**
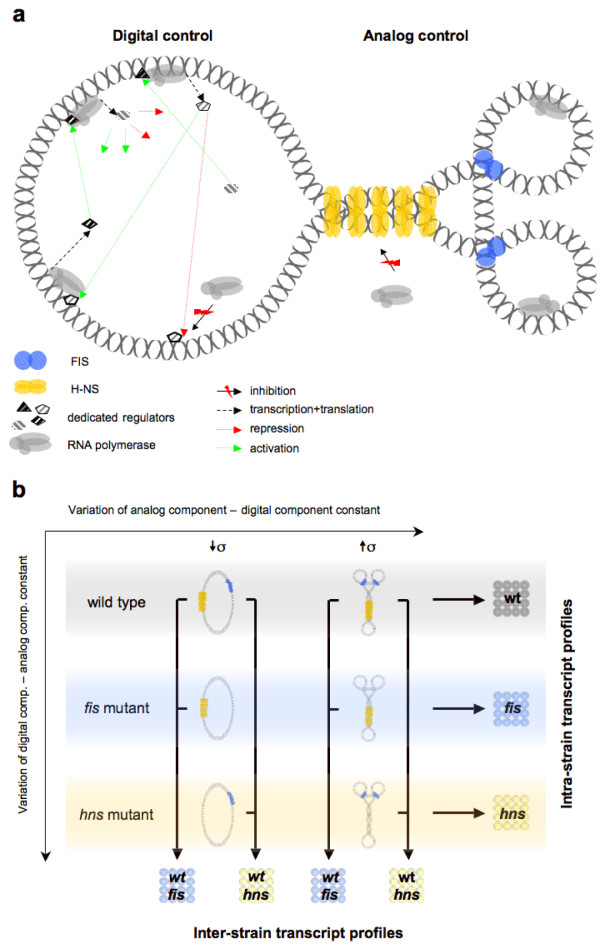
**Experimental design**. **(a) **Schematic representation of digital-type vs. analog-type of regulation. Digital control: Dedicated regulators independently recruit polymerase to distantly located genes to either activate (green arrows) or repress (red arrows) their activity. Analog control: Abundant DNA architectural proteins (only FIS and H-NS are shown for simplicity) form topological domains, thus rendering the distant genes under independent digital control similarly accessible to polymerase. The activation of transcription is indicated by RNA polymerase associated with DNA, repression of transcription by "red-flashed" arrows. **(b) **In our experimental setup the transcript profiles of three *E. coli *strains (wild-type, *fis *mutant, *hns *mutant) are compared under low (↓σ) and high superhelicity (↑σ) and also with each other (vertical connections). The three intra-strain transcript profiles (wt, *fis*, *hns*) show differentially expressed genes in response to variation of negative supercoiling but under a constant transcriptional regulatory network. The four inter-strain profiles (wt-*fis *and wt-*hns *for ↓σ and ↑σ each) show genes differentially expressed under constant supercoiling but with different genetic backgrounds. Note that alterations in superhelical density caused by mutations themselves are negligible compared to the experimentally induced changes of superhelicity [11].

In this study we aim at understanding the relationships between the digital and analog types of control in transcriptional regulation by using the model system of exponentially growing *E. coli *cells. The rationale is to investigate transcript profiles obtained under conditions where we either modulate the analog component of regulation under constant digital control, or modulate the digital component keeping the analog control constant. We modulate the analog component by experimentally varying the negative superhelical density (-σ) of chromosomal DNA within the same genetic background (i.e. with constant digital TRN). Such variation of -σ is carried out within three genetic backgrounds – the wild type *E. coli *and two mutant strains lacking one of the two abundant DNA architectural proteins, either FIS or H-NS. These comparisons produce the so-called intra-strain transcript profiles [11] (see Figure 1b). Modulation of the digital component (TRN) is achieved by mutating genes of the same two global DNA architectural proteins (either *fis *or *hns*, both representing hubs in the TRN) and comparing the wild type and mutant transcript profiles at a single constant superhelical density – either DNA relaxation (-σ < 0.033) or high negative supercoiling (-σ > 0.08). These comparisons produce the so-called inter-strain transcript profiles [11]. The first approach enables us to assess the impact of digital control in transcriptional regulation under variation of the analog component. The second approach allows us to assess the impact of analog control under variation of the digital component. We thus obtain seven data sets (Figure 1b): three distinct intra-strain transcript profiles reflecting digital-type control (wt, *fis*, *hns *for wild type, *fis *mutant and *hns *mutant backgrounds respectively), and four inter-strain profiles (wt-*fis *and wt-*hns *both at relaxation (↓σ) and high negative supercoiling (↑σ) reflecting analog-type control).

**Figure 2:**
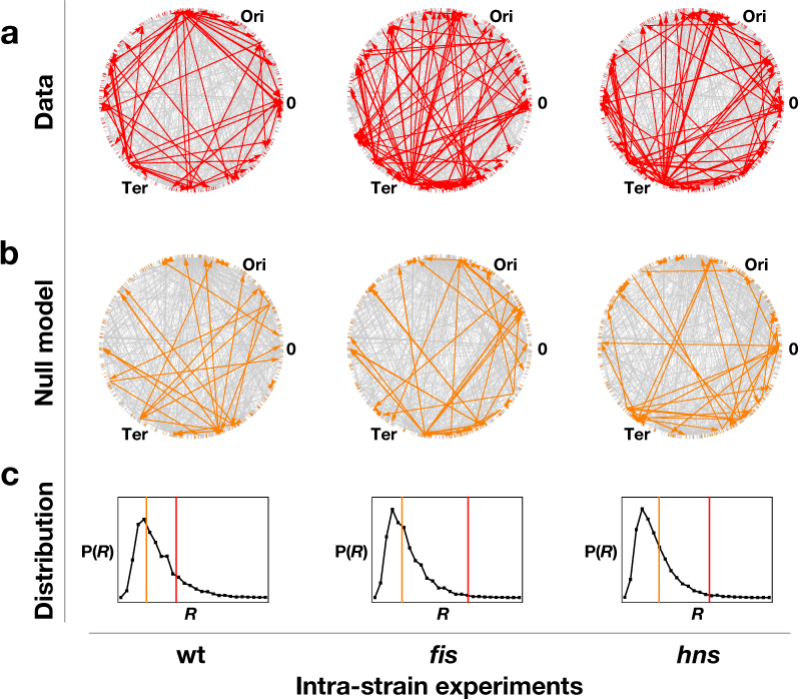
**Calculation of the digital control type confidence (CTC)**. (a) The effective TRNs (red) within the entire RegulonDB TRN (gray), mapped on the circular genome of *E. coli*. Only the three intra-strain experiments are shown. (b) The effective TRNs (orange) within the entire RegulonDB TRN (gray) for a single null model realisation. The effective TRNs of the null models are less densely connected. (c) The frequency distribution of *R *for 10,000 null models together with the actual values of *R *from the graphs shown in (a) and (b).

**Figure 3:**
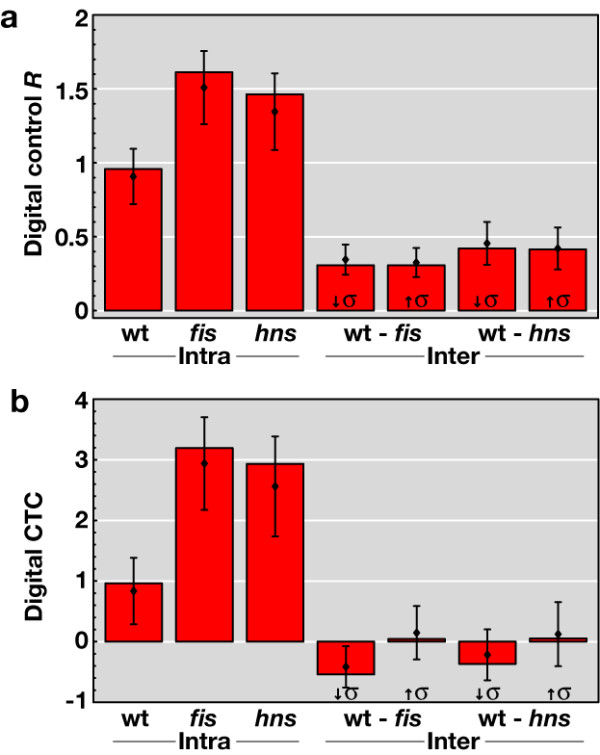
**Digital control *R *and control type confidence (CTC) of transcriptional regulation**. (a) The digital control *R *is the number of connected nodes divided by the number of isolated nodes in the effective transcriptional regulatory networks of the three intra-strain experiments (wt, *fis*, *hns*) and the four inter-strain experiments (wt-*fis *and wt-*hns *for low (↓σ) and high (↑σ) negative supercoling each). (b) Digital CTC quantifies the deviation of the effective subnet based on significant expression changes from an appropriate null model. To estimate the sensitivity of the observables against noise, we replace 10% of all affected genes with randomly selected genes from the pool of affected genes in all other experiments. We then recalculate the digital control via the ratio *R *(a) and the corresponding CTC (b). We show the mean *R*s (diamonds) together with the standard deviation for 10,000 runs, and the mean CTCs (diamonds) together with the standard deviation for 1,000 runs, where the actual data is compared to 1,000 null model runs each.

The transcriptional regulatory network (TRN) of E. coli is the basis of many recent studies on network architecture [14,15], as well as on the consistency of the network with expression profiles [6,7]. To assess the impact of digital-type control we analyze subnets of the TRN of *E. coli *spanned by genes with significantly changed expression in our three intra-strain transcript profiles, the *effective *TRNs (Figure 2). A convenient way of formalizing properties of these subnets is to analyze the ratio of genes with and without links, respectively. We define the control ratio *R *as the number of connected nodes divided by the number of isolated nodes in the effective TRN. Comparing this ratio with corresponding random models (see Figure 2) we obtain the z-score of this ratio, which we denote the control type confidence (CTC). The CTC quantifies how much above-random connectivity is found in the effective network and, consequently, how much control the network exerts on the expression profile. Formally, the digital CTC is the z-score of the control ratio *R *for the effective TRN, when compared to the distribution of control ratios, where the same number of affected nodes is mapped randomly on the TRN. We find a ratio *R *> 1 and CTC values beyond 2 only for two data sets – the intra-strain profiles of the *fis *and *hns *mutants (Figure 3), indicating that compared to wild type, in both mutants transcriptional regulation comprises a large proportion of digital-type control. Thus unexpectedly, mutations of global regulators, which represent hubs targeting disproportionately large numbers of genes in the TRN, increase rather than decrease the number links in the effective TRN and thus enhance digital control. At the same time, effective TRNs of the four inter-strain profiles did not deviate substantially from a random model (Figure 3b), as expected from our experimental design. This is because in the intra-strain profiles the constant digital control (background-specific TRN) enables to measure its impact under the variation of analog component (superhelical density σ), whereas in the inter-strain transcript profiles the TRN itself is a variable. The concept of an effective TRN thus allows quantifying the contribution of digital control to genomic expression patterns.

**Figure 4:**
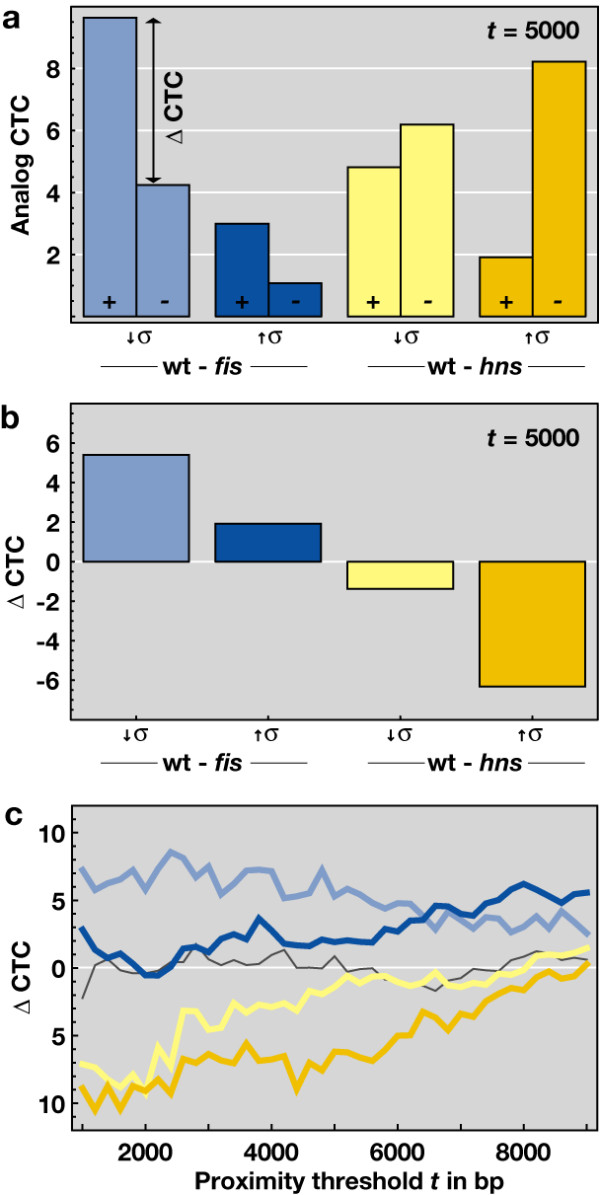
**Analog control-type confidence of gene proximity networks**. (a) Analog CTC of the four inter-strain expression profiles at proximity threshold t = 5 kb. The left (+) and right (-) bars correspond to expression data with log-ratios above and below 0, respectively. A positive log ratio (+) is associated with either a raised expression in wild type, or a inhibited expression in the mutant strain. (b) Difference (left bar – right bar) of the analog CTCs from (a) for each inter-strain experiment at *t *= 5 kb. (c) Difference of the CTCs for each inter-strain experiment against the proximity threshold *t*. For *t *> 10 kb, the effective GPNs are almost fully connected and a proper CTC calculation fails. Note that *fis *(blue) has a preponderantly activating and *hns *(yellow) a preponderantly repressing regulatory effect. As expected, the wild type intra-strain experiment does not deviate strongly form the random null model (dark gray) and results in ΔCTC ≈ 0.

The schematics used in Figure 1a represent the differences between the digital and analog types of control. In order to analyze the digital and analog types of control on the same methodological basis, we convert the chromosomal neighborhoods of genes into a network, designated the gene proximity network (GPN) (see Materials and Methods for details on the construction algorithm). The GPN subnet analysis of the inter-strain transcript profiles exposes the extent of spatial connectivity between the neighboring loci and reveals the clustering of significant expression changes in the genome. Alike the digital CTC, the analog CTC represents the z-score of the control ratio for the effective GPN, obtained in comparison to the distribution of control ratios for a null model, in which the same number of affected nodes is mapped randomly on the genome. An important difference between the FIS and H-NS effects on the three-dimensional structure of DNA is of note here. Although both are abundant DNA binding proteins occupying multiple chromosomal sites, H-NS is forming filaments by stabilizing tight DNA plectonemes and acting as a universal repressor for the bacterial genome, whereas FIS is stabilising toroids and superhelical loops implicated in activation of genes involved in metabolism and growth [[11,16-18]; see also the schematics depicting the different effects of FIS and H-NS on three-dimensional structure of DNA in Figure 1a]. Thus, directionally opposite effects – one largely of activation and another of global repression – are expected to underlie the GPNs in the inter-strain comparisons of wild type strain with *fis *and *hns *mutants, respectively. The GPNs of the *hns *mutant primarily reflect the spatial connectivity between de-repressed genetic loci, especially since H-NS represses whole regulatory systems rather than selectively targeted individual gene components [16,19]. We therefore assign to the wild type background the genes with positive log ratio in both *fis *experiments (wt-*fis *↑σ and ↓σ) and the genes with negative log ratio in both *hns *experiments (wt-*hns *↓σ and ↑σ). A consistent difference of calculated CTCs is observed over the whole sensible range of GPN thresholds (Figure 4c). In both inter-strain GPNs derived from the comparisons of wild type with *fis *mutant (wt-*fis *↓σ and ↑σ) in Figure 4a and 4b, the genes with a positive log ratio exhibit a higher CTC. As expected, an opposite result is obtained with *hns *mutant (wt-*hns *↓σ and ↑σ) in Figure 4a and 4b, where genes with a negative log ratio clearly exhibit a higher CTC. As expected for intra-strain profiles in which due to experimental design the analog component (i.e. the superhelical density -σ) itself is a variable, the GPN does not deviate strongly from random model (see dark gray curve in Figure 4c). Since besides their long range effects on three-dimensional structure of DNA, FIS and H-NS also appear as TRN hubs exerting specific effects on binding isolated gene promoters, we eliminated from our GPN analysis all the genes known to be specifically regulated by promoter binding of H-NS or FIS. However, this did not significantly affect the results of the GPN analysis (a change of the ΔCTC of less than 10% on average). Our GPN analyses thus indicate a high spatial connectivity of neighboring genes in wild type strains as opposed to strains lacking either FIS or H-NS. This high spatial connectivity depends on the superhelical density and its extent cannot be explained on the basis of the known specific binding sites of FIS and H-NS. Analysis of an operon based proximity network does not substantially alter the observed results (data not shown). We infer that the constraint of DNA supercoils and resultant reshaping of DNA by abundant bacterial nucleoid proteins FIS and H-NS substantially contributes to the analog-type of transcriptional control employing the spatial connectivity between neighboring genetic loci.

## Discussion

A unifying approach enabling to combine the data derived by different methodologies is essential for understanding the basic organizational principles of transcriptional regulation, especially since recently transcriptional sub-networks with organizationally distinct architectures have been described [20]. In this study we dissect the logical types of information derived by two established methodologies studying transcriptional regulation based either on TRN analyses, or on the analyses of transcriptional supercoiling response of genomic expression patterns. We denote the information retrieved by assessing directional interactions between the genes in TRN as digital, whereas we denote the information retrieved by assessing the influence of superhelical density on expression patterns as analog. This dissection enables us to present a generic approach allowing both, to distinguish and to assess the relationships between two logically distinct types of transcriptional control.

**Figure 5:**
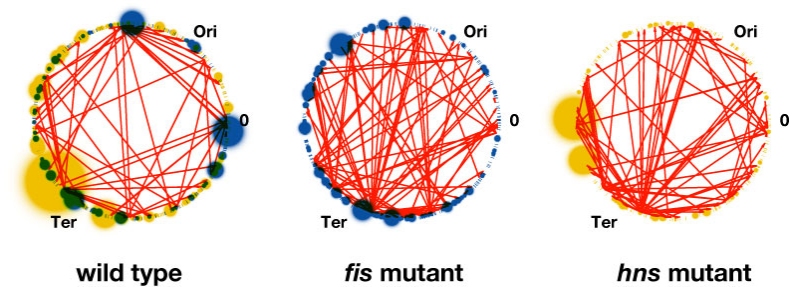
**Distribution of control**. The organizational logic of transcriptional regulation revealed by combining the information on digital control (obtained from intra-strain experiments), and information on analog control (obtained from inter-strain experiments). Red arrows indicate the links of the effective TRNs. Colored segments on the circular genome are affected genes, as derived from the *fis *(blue) and *hns *(yellow) inter-strain experiments. The colored spheres indicate connected components in the effective GPN at a proximity threshold of t = 5 kb. The actual size of the spheres is proportional to the diameter of the subnets spanned by each analyzed profile. The origin (Ori) and terminus (Ter) of chromosomal replication are also shown.

Using this approach we demonstrate that variation of the analog component of regulation (changing DNA superhelicity) effectively exposes the contribution of digital-type control (represented by the TRN) to transcriptional regulation, which is significantly increased in *E. coli *strains lacking global DNA architectural proteins. In turn, alterations of the digital component (changing TRN by deleting hubs) expose a substantial contribution of analog-type control (approximated by the GPN) to transcriptional regulation in wild type cells. Since the digital and analog types of control are constituents of a single transcriptional regulatory system of the cell, our data suggest that these two logically distinct types of control are balancing each other, such that upon limitations of one type of control (caused e.g. by mutation of a global DNA architectural factor) the other type can compensate for compromised regulation (Figure 5). What is the mechanism of compensatory increase of digital control observed in *fis *and *hns *mutant cells upon variation of superhelical density? The increased digital control may reflect a necessity of enhanced engagement of small regulatory circuits for sustaining cellular metabolism and growth, because the loss of global DNA architectural factors FIS and H-NS impairs the function of the overarching network of nucleoid-associated proteins and topoisomerases in regulating the supercoiling response of transcription [18].

While this network is intimately involved in spatial organization of transcription in E.*coli*, spatial organisation of transcription is observed in both, prokaryotes and eukaryotes [21,22]. In *E. coli *this phenomenon can be readily rationalized on the basis of topological domains of variable size underlying the organization of bacterial chromosome [23-25]. Indeed, both FIS and H-NS have been directly implicated in formation of topological barriers to supercoil diffusion [26]. Thus the preponderance of analog-type control in the wild type cells compared to mutants lacking FIS and H-NS (see Figure 5) is in keeping with the property of these proteins to stabilize supercoils and modulate the distributions of effective superhelicity in the genome [17,18]. Furthermore, observed alterations of spatial connectivity by mutations of *fis *and *hns *genes are also consistent with long-range effects of these proteins on the three-dimensional structure of DNA [16,18,25]. Finally, our GPNs analyses reveal that the control by FIS is more pronounced at low superhelical densities, whereas in the case H-NS the spatial control is more pronounced under conditions of high superhelical density (Figure 4c). These observations are fully consistent with the property of FIS to buffer upon DNA relaxation the activity of strong supercoiling-dependent promoters, such as those involved in ribosome production [18], whereas H-NS has been shown to predominantly repress the genes requiring high negative superhelicity during the exponential growth phase [11]. We note however, that these differences might also include some variations of mRNA stability. These effects are beyond the concepts (particularly the distinction between the two logical types of control) outlined here and may very well account for some of the expression changes not explained by the TRN or the GPN.

One prediction from the observed interdependence between digital and analog types of transcriptional control is that adaptive mutations in *E. coli *will affect the determinants of global DNA architecture. Indeed, a recent study of long-term experimental evolution in *E. coli *unmasking DNA topology as a key target for selection identified fitness-enhancing mutations in topoisomerase and *fis *genes [27]. Furthermore, such "evolved" populations possess high adaptational flexibility [3]. We propose that the buffering of transcriptional regulation by balancing effects of analog and digital types of control can counteract the reduction of adaptational flexibility caused by accumulation of mutations in bacteria [2]. In this respect it is revealing, that *fis *is a relatively late acquisition in bacterial evolution [28], whereas H-NS is implicated in regulating "adaptive" gene rearrangements and minimizing the cost of competitive fitness during horizontal gene transfer [19,29].

## Conclusion

We believe that the general concept of distinguishing logical types of control developed in this study will apply to many complex biological networks. We shall also emphasize that based on our data, reinterpretation of the interactions contained in the *E. coli *TRN database RegulonDB with respect to both, their digital and analog control characteristics – for example, consideration of the supercoiling sensitivity of the genes – might be a worthwhile extension of this database.

## Methods

### Microarray and network data

Transcript profiling for wild type, *fis *and *hns *LZ strains was carried out using *E. coli K12 V2 OciChip*™ DNA microarray. The genetically engineered *E. coli *LZ41 and LZ54 strains contain drug-resistant topoisomerase gene alleles enabling to selectively inhibit either DNA gyrase or topoisomerase IV activity and respectively induce either relaxation or high negative supercoiling [30]. The *fis *and *hns *mutants of the LZ41 and LZ54 strains were obtained by phage P1 transduction. Introduction of the *fis *and *hns *mutations in the LZ41 and LZ54 strains does not substantially alter the global supercoiling response to drug (norfloxacin) addition [11]. Each experiment was performed as two biological replicates with two technical replicates each, resulting in 28 cDNA microarray hybridisations. Scanned array images were quantified and normalized by applying a LOWESS (locally weighted scatterplot smoothing) algorithm to the data within print-tip groups using the TM4 software package [31]. A one-class t-test was applied to replicated experiments to obtain genes with significant changed expression. For all results presented in our article, we used a significance level α = 0.05. However, we find that the results remain unaffected over a wide range of significance levels (0.05 > α > 0.02). DNA microarray data sets have been deposited in the Array Express data bank with the accession number E-TABM-86. For detailed DNA microarray data description and analyses see [11].

The latest version of the RegulonDB 5.6 data sets [4] "gene product" [32] and "regulatory network interactions" [33] were used for gene proximity network (GPN) and transcriptional regulatory network (TRN) generation, respectively.

### TRN construction

Preceding the construction of effective TRNs, dimeric regulatory gene identifiers in the microarray data (*flhC, flhD; gatR_1, gatR_2; hupA, hupB; ihfA, ihfB; rcsA, rcsB*) were replaced by unique Regulon DB identifiers (*flhCflhD; gatR_1gatR_2; hupAhupB; ihfAihfB; rcsArcsB*). The effective TRN subnet of a DNA microarray transcript profile is the set of affected genes in the TRN and their regulatory interactions contained in RegulonDB (see Additional file 1 for edge lists of the resulting effective TRNs). Connected components of an effective TRN emerge, if both regulating and regulated genes are affected in the transcript profile (see subnet analysis and Figure 2). Connected and unconnected subnet components were further analysed [see Additional file 1].

### GPN construction

Preceding GPN subnet construction, the inter-strain transcript profile data was split up into genes with positive and negative log ratios, respectively. Genes with positive log ratios refer to high transcript levels in wild type background, genes with negative log ratios refer to high transcript levels in *fis *or *hns *mutant background. GPN subnets of the split DNA microarray transcript profiles were generated based on genomic position of affected genes together with the proximity threshold *t*, given in in nucleotide bases (b). All affected genes with spatial distance (here distance is relating to ORF start and stop position) below the selected proximity threshold *t *were considered as connected. GPN subnets were generated for a meaningful range of 1b <*t *< 10 kb, resulting in connected genes within an operon scale at *t *≈10b, up to completely conntected GPNs for *t *> 10 kb. Connected and unconnected subnet components were further analysed [see Additional file 2].

### Subnet analyses

For each subnet, the control ratio *R *was calculated as the number of connected nodes *N*_connected _(i.e. the size of the connected subnet component) over the number of isolated nodes *N*_isolated _(i.e. the size of the unconnected subnet component), *R *= *N*_connected_/*N*_isolated_. The control type confidence, CTC, is the z-score of *R*, calculated from the mean *R *and its standard deviation obtained from 10000 runs of the corresponding null model. In the case of the digital null model, the same number of affected nodes was mapped randomly on the TRN (see Figure 2). For the analog null model, the same number of affected genes was mapped randomly on the positions in circular genome.

The robustness of calculated ratios and CTCs was verified by 10% random data replacement with data of all affected genes from the remaining DNA microarray sets (see Figure 3).

## Abbreviations

CTC, control type confidence, GPN, gene proximity network, TRN, transcriptional regulatory network.

## Authors' contributions

CM, MG, MTH, and GM conceived the study. CM and MG analyzed the data. CM, MG, MTH, and GM wrote the paper. All authors read and approved the final manuscript.

## Supplementary Material

Additional file 1 Dataset S1. Gene identifier and corresponding edge lists of the seven directed effective TRNs emerging from the analysis of the seven transcript profiles. Click here for file

Additional file 2 Dataset S2. Gene identifier and corresponding edge lists of the eight undirected GPNs emerging from the seperated analysis of the four inter-strain transcript profiles with positive and negative log ratio, respectively. Click here for file
